# Does Motor Memory Reactivation through Practice and Post-Learning Sleep Modulate Consolidation?

**DOI:** 10.3390/clockssleep5010008

**Published:** 2023-02-17

**Authors:** Whitney Stee, Philippe Peigneux

**Affiliations:** 1 UR2NF—Neuropsychology and Functional Neuroimaging Research Unit Affiliated at CRCN—Centre for Research in Cognition and Neurosciences and UNI—ULB Neuroscience Institute, Université Libre de Bruxelles (ULB), 1050 Bruxelles, Belgium; 2 GIGA—Cyclotron Research Centre—In Vivo Imaging, University of Liège (ULiège), 4000 Liège, Belgium

**Keywords:** reactivation, retrieval, sleep, motor memory, memory consolidation

## Abstract

Retrieving previously stored information makes memory traces labile again and can trigger restabilization in a strengthened or weakened form depending on the reactivation condition. Available evidence for long-term performance changes upon reactivation of motor memories and the effect of post-learning sleep on their consolidation remains scarce, and so does the data on the ways in which subsequent reactivation of motor memories interacts with sleep-related consolidation. Eighty young volunteers learned (Day 1) a 12-element Serial Reaction Time Task (SRTT) before a post-training Regular Sleep (RS) or Sleep Deprivation (SD) night, either followed (Day 2) by morning motor reactivation through a short SRTT testing or no motor activity. Consolidation was assessed after three recovery nights (Day 5). A 2 × 2 ANOVA carried on proportional offline gains did not evidence significant Reactivation (Morning Reactivation/No Morning Reactivation; *p* = 0.098), post-training Sleep (RS/SD; *p* = 0.301) or Sleep*Reactivation interaction (*p* = 0.257) effect. Our results are in line with prior studies suggesting a lack of supplementary performance gains upon reactivation, and other studies that failed to disclose post-learning sleep-related effects on performance improvement. However, lack of overt behavioural effects does not detract from the possibility of sleep- or reconsolidation-related covert neurophysiological changes underlying similar behavioural performance levels.

## 1. Introduction

The formation of new memories is a complex temporal phenomenon undergoing successive stages before stabilization/consolidation [1]. Following the encoding phase, structural transformations are needed to enable strengthening and stabilization of the initial traces at the synaptic level [2,3]. Newly created engrams are subsequently reorganized over extended periods of time within memory type-specific neural networks [4,5]. Once the process is successfully completed, information can be retrieved to generate behavioural outputs [6]. However, retrieving information actually reactivates the memory trace, subjecting it again to a labile state, potentially sensitive to interference and/or reinforcement [7,8,9,10]. After being put in a reactivated/labile state during retrieval, a new protein synthesis step [6] is needed to restabilize the updated memory in a strengthened [11,12,13,14] (if the original trace was reinforced) or weakened [8,15,16,17] (if exposed to interfering material) form during the reconsolidation process [8,18]. Likewise, post-learning sleep has been shown to play a specific role in the delayed reorganization of neural networks [19,20] and is proposed to actively promote long-term consolidation processes through continued learning-related brain activity, i.e., neuronal replay [21]. In the motor domain, experience-dependent reactivation in learning-related brain areas was evidenced in man during NREM [22] and REM [23,24] sleep. Additionally, performance improvement on the next day was determined to correlate with the magnitude of regional brain activity during post-learning sleep [25], suggesting a role for sleep-related reactivation in memory retention processes. Neuronal replay and synaptic growth after motor learning have also been observed in rodents during subsequent NREM sleep [26,27], and brain gene expression after sensorimotor experience during REM sleep [28]. Delayed reactivation after sleep and/or time thus appears to play a role in post-learning modifications (i.e., weakening or strengthening) of consolidated memory traces.

Motor sequence learning is known to follow two main steps in the evolution of performance. First, a rapid increase in speed and accuracy during actual, online learning of the motor sequence. Second, a spontaneous, offline increase in performance parameters that develops over post-learning time intervals (i.e., outside of actual motor practice), corresponding to the consolidation phase [29,30] during which practice-triggered neural processes continue unfolding. Although the role of post-training sleep in motor memory consolidation processes has been extensively studied, a consensus is not reached yet [31]. Whereas some studies highlight a beneficial effect of post-training sleep on motor memory consolidation [32,33,34,35], others did not evidence post-training sleep-related effects on the development of offline behavioural gains [36,37,38,39]. Meta-analytic reviews agree on a globally positive effect of post-training sleep on the evolution of behavioural performance [31,40] with small size effects [31].

So far, the necessity to use invasive experimental techniques restricted reconsolidation studies mostly to animal populations [6,41]. Still, several studies investigated reconsolidation triggered by motor memory retrieval in humans using non-invasive protocols (for a review, see [42]). For example, the primary motor cortex (M1) appears to play a particular role in the reorganization of the motor memory trace; repetitive Transcranial Magnetic Stimulation (rTMS)-induced inactivation of M1 during reactivation hampers further performance gains [17]. At the behavioural level, it was shown using a sequential Finger Tapping Task (FTT; [29]) that a short reactivation of the initially learned sequence immediately before practice of a novel interfering sequence deteriorates performance at the subsequent retrieval of the original sequence [15]. It indicates that motor memory reactivation through retrieval is sufficient to make the initial memory trace transiently labile and sensitive to interference, an effect modulated by the length of the reactivation [43] and the delay between reactivation and interferent tasks [44]. Interference effects after reactivation in man have been confirmed using non-invasive behavioural [45,46] and brain stimulation techniques [16,17]. On the other hand, motor memory reactivation can also lead to motor performance improvement if the learned material is reinforced rather than interfered with [13,14]. For instance, retraining participants extensively on FTT or over a short period of time but with an outstanding continuity, gave rise to improved delayed performance [13]. In addition, exposing volunteers to a slightly modified version of a Sequential Visual Isometric Pinch Task [47], including minor motor-sensory variations as compared to the learning task, was determined to boost performance [14].

**Figure 1:**
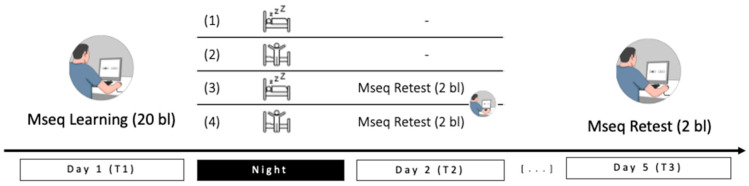
Experimental design and groups: Participants were trained on Day 1 in the late afternoon on 20 SRTT blocks, then groups 1 and 3 were allowed to sleep for the night (RS) while groups 2 and 4 spent the night awake (SD). In the morning of Day 2, groups 3 and 4 performed a 2-block retest (Reactivation condition). After three nights of sleep at home, all groups came back to the lab for a final 2-block retest (Day 5). Mseq: motor sequence task.

Evidence gathered so far suggests that active reactivation during wakefulness through practice (e.g., during testing) on the learned material may trigger reconsolidation processes, possibly leading to the strengthening of the memory trace and inducing further gains in performance. Concomitantly, post-training sleep and spontaneous reactivation of motor memories during sleep can help stabilizing newly formed memory traces that might become, in this case, less modulable following practice-related reactivation. However, few studies investigated motor memory improvement through behavioural trace reactivation, and to our knowledge, none investigated its potential interaction with sleep. In the present study, we investigated the effect of a short behavioural reactivation episode (vs. none) on the morning after post-training sleep (vs. sleep deprivation) on delayed motor performance after three nights of recovery sleep using a motor sequential Serial Reaction Time Task (SRTT—[48]; see Figure 1). Besides main practice-related reactivation and post-training sleep effects on performance improvement evidenced in prior studies, we hypothesized that the availability of post-training sleep may dampen the effect of morning reactivation on delayed performance after three supplementary post-reactivation nights.

## 2. Results

### 2.1. Demographic Data

Separate ANOVAs conducted on laterality, sleep quality and age with between-subject factor *Group* (1, 2, 3, 4) were non-significant (all *ps* > 0.343; 0.151 > BF_incl_ > 0.086). Regarding the chronotype, the ANOVA revealed a significant *Group* effect (F_3, 40.609_ = 2.846, *p* = 0.049, *η*_p_^2^ = 0.088, Bayesian Factor [BF_incl_] = 0.864). However, when conducting post hoc comparisons, none of the pairs was significant (all *ps* > 0.111).

### 2.2. Learning Session

**Figure 2:**
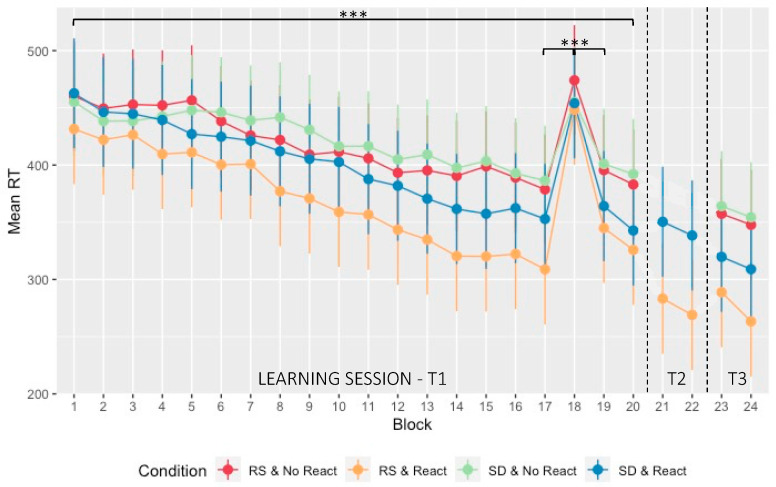
Performance evolution (speed): Mean RT (in msec) at the SRTT plotted over the different testing days and blocks with standard deviation. The learning session consisted of 20 sequential blocks (blocks 1 to 20 with block 18 pseudo-random—T1). On the next morning and following the experimental night (regular sleep (RS)/sleep deprivation (SD)), a short reactivation took place on two blocks (blocks 21 and 22—T2) for the Morning Reactivation groups (React) only. After three nights of recovery sleep at home, performance was assessed for all groups on two last blocks (blocks 23 and 24—T3). *** *p* < 0.001. *RS & No React* (red) = regular sleep and no Morning Reactivation; *RS & React* (orange) = regular sleep with Morning Reactivation; *SD & No React* (green) = sleep deprivation and no Morning Reactivation; *SD & React* (blue) = sleep deprivation with Morning Reactivation.

An ANOVA was computed on mean reaction time (RT) for sequential blocks with within-subject factor *Block* (1:17, 19:20) and between-subject factors *Sleep* (RS/SD on the experimental night) and *Reactivation* (Morning Reactivation/No Morning Reactivation). The ANOVA disclosed a main *Block* effect with a progressive decrease in mean RT (F_3.831, 287.344_ = 52.783, *p* < 0.001, *η*_p_^2^ = 0.413, BF_incl_ = 2.219 × 10^13^; Figure 2). As expected at the pre-experimental manipulation session stage, the main *Sleep* (F_1, 75_ = 0.899, *p* = 0.346, *η*_p_^2^ = 0.012, BF_incl_ = 0.352) and *Reactivation* effects (F_1, 75_ = 3.611, *p* = 0.061, *η*_p_^2^ = 0.046, BF_incl_ = 1043.895) were non-significant. However, there was a significant Block*Reactivation interaction effect (F_3.831, 287.344_ = 3.219, *p* = 0.014, *η*_p_^2^ = 0.041, BF_incl_ = 2399.488), with a larger RT improvement in the Morning Reactivation than in the No Morning Reactivation conditions. No other interaction effect was significant (all *ps* > 0.470, 0.537 > BF_incl_ > 7.558 × 10^−7^). Hence, and unexpectedly, RT performance evolution in the learning session was steeper in the Morning Reactivation than in the No Morning Reactivation condition. Consequently, further analyses on between-sessions performance improvement were computed on proportional changes from the end of the learning Day 1 session.

Separate ANOVA comparing sequential (blocks 17, 19) and pseudo-random (block 18) conditions showed that RT in the 18th block was significantly slower than in the 17th (*p* < 0.001) and 19th blocks (*p* < 0.001), suggesting that participants anticipated the upcoming position in sequential blocks (1:17, 19:20), i.e., learned the sequence, and that performance improvement was not merely due to motor practice (main *Block* effect: F_1.716, 128.681_ = 103.610, *p* < 0.001, *η*_p_^2^ = 0.580, BF_incl_ = 1.237 × 10^14^). In line with the ANOVA result reported above, the Block*Reactivation interaction effect was significant (F_1.716, 128.681_ = 3.656, *p* = 0.035, *η*_p_^2^ = 0.046, BF_incl_ = 2.288) and the main *Reactivation* effect (F_1, 75_ = 3.983, *p* = 0.050, *η*_p_^2^ = 0.050, BF_incl_ = 1.736) was marginally significant. No other effect reached significance (all *ps* > 0.101; 0.519 > BF_incl_ > 0.109).

**Figure 3:**
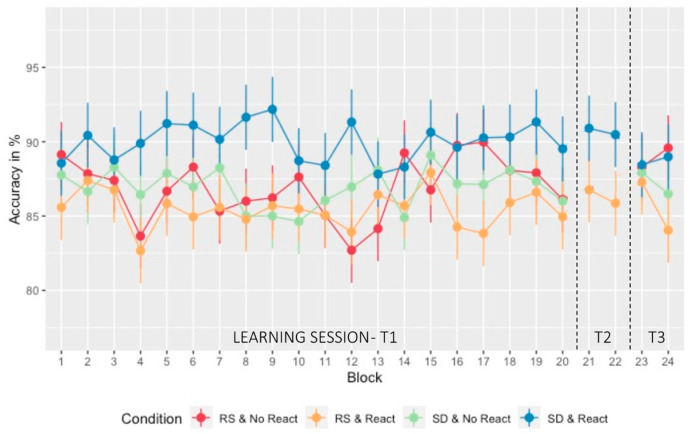
Performance evolution (accuracy): Percentage of correct triplets at the SRTT with standard deviation over the different testing days and blocks. The learning session consisted of 20 sequential blocks (blocks 1 to 20 with block 18 pseudo-random—T1). On the next morning and following the experimental night (regular sleep (RS)/ sleep deprivation (SD)), a short reactivation took place on two blocks (blocks 21 and 22—T2) for the two Morning Reactivation groups (React) only. After three nights of recovery sleep at home, performance was assessed for all participants on two last blocks (blocks 23 and 24—T3).

A similar ANOVA was conducted on accuracy scores with within-subject factor *Block* (1:18, 19:20) and between-subject factors *Sleep* (RS/SD on the experimental night) and *Reactivation* (Morning Reactivation/No Morning Reactivation). Accuracy remained stable over the learning session (Figure 3) with no main *Block* (F_11.371, 852.811_ = 1.030; *p* = 0.418, *η*_p_^2^ = 0.014, BF_incl_ = 7.181 × 10^−5^), *Sleep* (F_1, 75_ = 3.411, *p* = 0.069, *η*_p_^2^ = 0.043, BF_incl_ = 0.367), or *Reactivation* (F_1, 75_ = 0.525, *p* = 0.471, *η*_p_^2^ = 0.007, BF_incl_ = 0.126) nor any interaction (all *ps* > 0.067, 0.200 > BF_incl_ > 5.110 × 10^−12^) effects. A separate analysis disclosed no difference in accuracy in block 18 when compared to sequential block 17 or 19 (*p* = 0.887).

### 2.3. Difference in Performance at T2

Proportional offline gains were calculated as the difference between the mean of the two morning retest blocks (21:22—T2) and the mean of the two last learning session blocks (19:20—T1) divided by the mean of the two last learning session blocks (19:20—T1) and multiplied by 100 ($\frac{T3-T1}{T1}\times 100$). The Mann–Whitney U-test comparing proportional offline gains in Mean RT at the outset of the experimental sleep (RS) or sleep deprivation (SD) night for both Morning Reactivation groups revealed significantly less decrease in Mean RTs in SD (−1.858) compared to the RS (−18.242) participants (U_38_ = 67.000; *p* < 0.001; Cohen’s d = −0.665) whereas the Student *t*-test on accuracy showed no difference between groups (t_38_ = −0.022; *p* = 0.983; Cohen’s d = −0.007).

### 2.4. Motor Memory Consolidation Modulated by Post-Training Sleep and Reactivation Conditions

**Figure 4:**
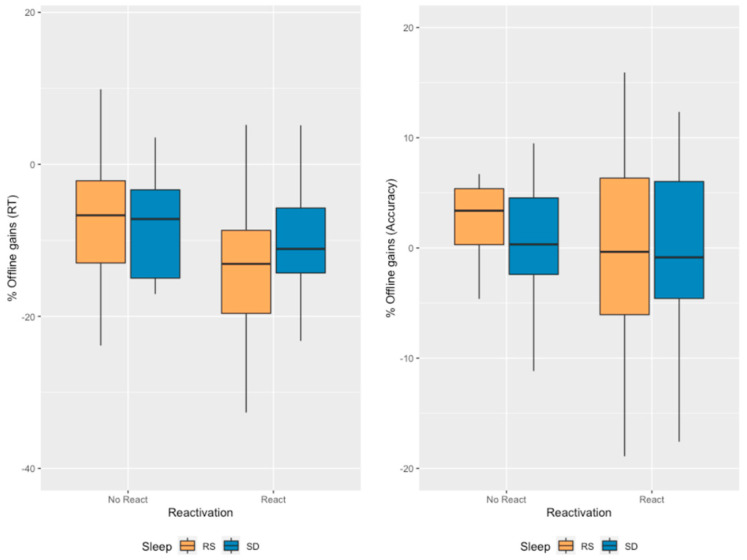
*Offline gains in performance.* The offline gains were computed by subtracting the mean of blocks 23 and 24 (T3) to the mean of the two last blocks of the LS (thus, blocks 19 and 20—T1) divided by the mean of the two last blocks of the LS before multiplying the score by 100 to obtain a percentage. No significant Reactivation, Sleep or interaction effects are evidenced.

To control for the fact that performance at the end of the Learning session was significantly different between the Reactivation and No Reactivation conditions, we computed proportional offline gains as the difference between the mean of the two final retest blocks (23:24—T3) and the mean of the two last learning session blocks (19:20—T1) divided by the mean of the two last learning session blocks (19:20—T1) and multiplied by 100 ($\frac{T3-T1}{T1}\times 100$). The ANOVA carried on proportional offline gains in mean RT with between-subject factors *Sleep* (RS/SD on the experimental night) and *Reactivation* (Morning Reactivation/No Morning Reactivation) did not disclose any significant *Reactivation* (F_1, 75_ = 2.813, *p* = 0.098, *η*_p_^2^ = 0.036, BF_incl_ = 0.588), Sleep (F_1, 75_ = 1.085, *p* = 0.301, *η*_p_^2^ = 0.014, BF_incl_ = 0.308) or Sleep*Reactivation interaction (F_1, 75_ = 1.303, *p* = 0.257, *η*_p_^2^ = 0.017, BF_incl_ = 0.308) effects (Figure 4). Bayesian statistics globally indicate robust evidence towards the null hypothesis (i.e., suggesting *Sleep* and Sleep*Reactivation do not influence mean RT). Concerning *Reactivation*, evidence remains unconclusive.

A similar ANOVA was conducted on proportional offline gains in accuracy with between-subject factors Sleep (RS/SD on the experimental night) and Reactivation (Morning Reactivation/No Morning Reactivation). The analysis revealed no significant main Reactivation (F_1, 75_ = 1.356, *p* = 0.248, *η*_p_^2^ = 0.018, BF_incl_ = 0.300), main Sleep (F_1, 75_ = 0.740, *p* = 0.392, *η*_p_^2^ = 0.010, BF_incl_ = 0.232) or Sleep*Reactivation interaction (F_1, 75_ = 7.550 × 10^−4^, *p* = 0.978, *η*_p_^2^ = 1.007 × 10^−5^, BF_incl_ = 0.091) effects (Figure 4). Bayesian statistics globally indicate robust evidence towards the null hypothesis (i.e., suggesting our factors do not influence accuracy).

### 2.5. Additional Control Analyses

#### 2.5.1. Alertness before Learning and Testing Sessions

An ANOVA with between-subject factor *Group* (1, 2, 3, 4) and within-subject factor *Time* (T1-Learning session, T3-Final retest) was computed on Reciprocal Reaction Time (RRT) in the PVT_5_ (1/RT [49]). The analysis indicated higher alertness before the retest on Day 5 than before the Learning session (F_1, 75_ = 4.598, *p* = 0.035, *η*_p_^2^ = 0.058, BF_incl_ = 0.885). However, there were no main *Group* (F_3, 75_ = 0.571, *p* = 0.636, *η*_p_^2^ = 0.022, BF_incl_ = 0.191) or *Time×Group* interaction (F_3, 75_ = 0.728, *p* = 0.538, *η*_p_^2^ = 0.028, BF_incl_ = 0.071) effects.

#### 2.5.2. Sleep Quality and Duration on the Experimental RS Night

ANOVAs were computed on sleep duration and sleep quality for the experimental RS night (derived from sleep logs) with between-subject factor *Group* (No Reactivation [1] vs. Reactivation [3]). No significant difference was determined between the two groups both for sleep duration (mean duration ± SD = 7.86 ± 0.97, min = 5.92, max = 10.33; F_1, 37_ = 1.734, *p* = 0.196, *η*_p_^2^ = 0.045, BF_incl_ = 0.616) and sleep quality (mean score ± SD = 517.76 ± 62.53, min = 369.17, max = 612.50; F_1, 37_ = 0.036, *p* = 0.851, *η*_p_^2^ = 9.647 × 10^−4^, BF_incl_ = 0.316).

#### 2.5.3. Sleep Quality and Duration during the Three Recovery Nights

A mixed ANOVA was computed on sleep duration from the three recovery nights (derived from sleep logs) with within-subject factor *Night* (4th, 5th, 6th) and between-subject factors *Sleep* (RS/SD on the experimental night) and *Reactivation* (Morning Reactivation/No Morning Reactivation). A main *Night* effect (F_1.843, 138.190_ = 6.751, *p* = 0.002, *η*_p_^2^ = 0.083, BF_incl_ = 8.797) was determined with a significantly longer sleep duration (*p* = 0.005) on the 4th night compared to the 5th and the 6th night. In addition, a main *Sleep* effect (F_1, 75_ = 6.877, *p* = 0.011, *η*_p_^2^ = 0.084, BF_incl_ = 1.513) revealed to be significant with a higher sleep duration in both SD groups compared to the RS groups (*p* = 0.011). However, no other effect was revealed to be significant (all *ps* > 0.194, 0.551 > BF_incl_ > 0.006).

Concerning sleep quality, a similar ANOVA was performed. However, no significant difference was detected (all *ps* > 0.194, 0.181 > BF_incl_ > 1.129 × 10^−4^).

#### 2.5.4. Vigilance and Sleepiness throughout the Experimental SD Night

An ANOVA performed on sleepiness KSS-scores with between-subject factor *Group* (No Reactivation [2] vs. Reactivation [4]) and within-subject factor *Time* (hourly score between 22h and 6h) evidenced a *Time* effect (F_4.023, 88.503_ = 13.855, *p* < 0.001, *η*_p_^2^ = 0.386, BF_incl_ = ∞) with increasing sleepiness all over the night. The main *Group* (F_1, 22_ = 0.006, *p* = 0.940, *η*_p_^2^ = 2.650 × 10^−4^, BF_incl_ = 0.415) and the *Time×Group* interaction (F_4.023, 88.503_ = 1.290, *p* = 0.280, *η*_p_^2^ = 0.055, BF_incl_ = 0.485) were non-significant.

Similarly, an ANOVA was performed on vigilance RRT (PVT_10_) scores with between-subject factor *Group* (No Reactivation [2] vs. Reactivation [4]) and within-subject factor *Time* (bi-hourly score between 22h and 6h). The analysis evidenced a *Time* effect (F_2.954, 112.266_ = 20.954, *p* < 0.001, *η*_p_^2^ = 0.355, BF_incl_ = 6.011 × 10^10^) with decreasing alertness over the night. *Group* (F_1, 38_ = 0.208, *p* = 0.651, *η*_p_^2^ = 0.005, BF_incl_ = 0.331) and *Time×Group* interaction (F_2.954, 112.266_ = 1.140, *p* = 0.336, *η*_p_^2^ = 0.029, BF_incl_ = 0.185) effects were non-significant.

## 3. Discussion

In this experiment, we aimed at investigating the influence of a short behavioural reactivation after post-training sleep or sleep deprivation on delayed motor performance. To this end, we manipulated in a between-groups 2 × 2 design the sleep opportunity on the experimental post-learning night (Regular Sleep (RS) vs. Sleep Deprivation (SD)) and the possibility for a reactivation through a short testing (two blocks) in the morning following the experimental night (Reactivation vs. No Reactivation conditions) in a 5-day protocol. We hypothesized that morning reactivation on post-learning Day 2 through a short SRTT retest would increase delayed behavioural gains at Day 5 and positive effect of post-training sleep on delayed offline gains. Additionally, we reasoned that consolidation during post-training sleep may dampen the effect of next morning’s reactivation on delayed performance. Contrary to our hypotheses, our results could not evidence a positive effect of post-learning Day 2 morning reactivation on delayed performance at Day 5. In addition, although performance improved at the delayed Day 5 session in all conditions, the offline gains were not modulated by the post-learning sleep opportunity, and there was no sleep by reactivation interaction effect, hence suggesting a mere time-dependent effect on the evolution of offline performance. 

As previously mentioned, the literature concerning motor memory strengthening by means of destabilization–reconsolidation remains scarce, in opposition to interferent effects that are more documented. Wymbs et al. [14], studying motor skill improvement through retraining, determined that the highest performance improvement was observed in a group retrained on a slightly modified version of their SVIPT task (as compared to the initial version), whereas there was no difference between a group retrained on the same version of the task and another not retrained at all. Hence, introducing minor motor-sensory variation might be necessary to boost performance through reconsolidation, which is in line with other studies suggesting that reconsolidation may be only initiated when updating is necessary [50], as this is believed to activate neural processes related to prediction error [51], a mechanism that seems to be efficient to cause motor memory updating and strengthening through reconsolidation [52]. Another study [13] investigated the effect of motor reactivation duration and consistency on delayed performance using a finger tapping task (FTT). The study determined that long reactivation (such as initial learning) benefited retention more than brief reactivations (30 s). Interestingly, it also determined that brief, continuous and error-free reactivations induced significant performance gains (comparable to the effect of extended practice), whereas participants exhibiting low continuity reactivations (i.e., with more frequent errors) were not different from control participants (no reactivation). In addition, while continuity predicted reactivation-associated offline gains, the mere number of errors itself did not, suggesting that the correct form of the memory trace needed to be reactivated with a minor impact from errors situated in the beginning or at the end of the task compared to errors made midway into the retrieval session that would completely undermine reactivation-related beneficial effect [13]. Thus, although reactivation of the memory trace appears to be an interesting tool to strengthen memory by triggering reconsolidation, it seems to be efficient only under strict conditions. In our case, the trace reactivation was identical to the initial learning task, meaning no discrepancy existed between predicted and actual outcomes. In addition, even though short reactivations were determined sufficient to destabilize a memory trace [13,44] and trigger reconsolidation processes, a longer retest might have been needed for our task. On the other hand, longer reactivation time may rather result in supplementary learning, preventing from isolation of the effect or the destabilization–reconsolidation process. In addition, the discrepancy between our results and the previous literature might also simply be due to task differences. Indeed, the recruitment of distinct brain areas and the need for developing various abilities has already been proposed to explain discrepant outcomes in the motor skill literature [40]. Lastly, other studies using motor tasks simply failed to induce reconsolidation through reactivation [53].

Considering the lack of sleep effect on delayed performance, our results are in line with other studies showing that post-learning sleep does not always result in increased offline gains [36,37,38,39]. Indeed, while sleep seems to have a general positive effect according to different meta-analysis [31,40], several factors have been hypothesized to modulate this effect such as, again, the task used in the paradigm [40]. When looking at experimental designs using SRTT, sleep-related effects are not consistently detected. While some studies show differentiated neural patterns between sleep and wake group 3 days after the experimental night with no behavioural differences [54], others fail to detect sleep-specific effects on the optimization of sequential components [55,56,57]. In addition, the awareness and learning intention from the participants seemed to modulate the effect of sleep as offline gains were only sleep-dependent in a more explicit condition, where the volunteers intentionally learned the motor sequence [58]. Lastly, Schönauer et al. [34] determined that participants who were sleep-deprived on the post-learning night were able to catch up and reach performance levels similar to those of the sleep group after 2 nights of recovery sleep (thus, 72 h post training), even though they did not show initial increase in performance right after the SD night (thus, 12 h post training) when compared to the regular sleep group. Thus, sleep-related offline gains might also have been compensated by further time and sleep as our final performance assessment took place 4 days after initial learning.

We did choose to target the mid-luteal phase instead of the follicular phase as prior studies indicated that women show a motor performance and sleep-related procedural memory consolidation similar to those of men in the mid-luteal phase in contrast to the follicular phase [59,60]. Concerning the premenstrual syndrome [PMS], symptoms seem to appear 6 days before menses onset and tend to peak 2 days before [61]. Our female participants underwent their learning session between days 17 and 21, automatically placing the last retest between days 21 and 25. PMS would start on day 23 and peak at day 27 with menses starting on day 1 of the next 28-day cycle. Considering the partial overlap between the beginning of PMS and the last retest, we decided to prioritize the similarity between men and women concerning motor memory consolidation, especially as PMS seems to significantly impact only 5 to 20% of the female population [61].

Admittedly, our study also contains certain limitations. An unexplained pre-experimental manipulation difference in performance was detected at the end of the learning session between our Morning Reactivation and No Morning Reactivation groups. We attempted to compensate for this unexpected discrepancy by analysing proportional differences in performance between the end of the learning session and the final retest. Nonetheless, we cannot rule out that such difference during the online acquisition of the sequence may trigger a different time course for offline consolidation and, potentially, reconsolidation.

In conclusion, more research is required to better understand the exact mechanisms and conditions needed for memory strengthening to appear following reconsolidation in humans. For the moment, only limited research was conducted considering the recent possibility to approach this topic through non-invasive approaches. In addition, further research nurturing the debate concerning the exact circumstances wherein sleep benefits memory is strongly needed.

## 4. Materials and Methods

### 4.1. Participants

Eighty healthy young volunteers (40 females) aged 18–30 years (mean age ± SD = 21.7 ± 2.68 years, min = 18, max = 30) provided written informed consent to participate in this study approved by the ULB-Erasme Ethics Committee. One was excluded due to short sleep duration on the experimental night (3 h). The RS with Morning Reactivation group consisted of 19 participants, while the 3 others included 20 subjects each. Computer scientists and musicians who might exhibit high-level hand dexterity, smokers, and individuals with neurological/psychiatric medical records or exposed to jetlag within the past 3 months were excluded. Participants had a moderate to neutral chronotype (mean score ± SD = 51.1 ± 7.82, min = 32, max = 66 at the Morningness–Eveningness Questionnaire [62]) and good sleep quality (score ≤ 7 at the Pittsburgh Sleep Quality Index [63], mean score ± SD = 3.78 ± 1.61, min = 0, max = 7). Both right- and left-handers were included due to the bimanual character of the chosen task (adapted from the Edinburgh Inventory [64], mean score ± SD = 8.06 ± 3.58, min = −9, max = 10).

### 4.2. General Procedure

Participants were pseudo-randomly assigned to one out of 4 groups (see Figure 1); gender was balanced within each group. Female participants were tested during their luteal phase to prevent a hormonal bias on motor performance and consolidation [59,65]. All participants were explicitly asked to refrain from drinking caffeine or other stimulating drinks on the testing days, and to keep a regular sleep schedule for the duration of the experiment (starting from 3 days before the learning session until the last retest). The regularity of their sleep–wake schedule was controlled from 3 days before the learning session until the end of the procedure using actimetric recordings (Actigraph^TM^ wGT3X-BT, Pensacola, FL, USA) and self-reported morning sleep logs (St. Mary’s Hospital sleep questionnaire [55]). 

At Day 1, participants came to the lab in the late afternoon for a 20-block learning session (approximate duration: 40 min) on the Serial Reaction Time task (SRTT; see Section 4.3. *Motor task* below). Each of the 20 blocks featured a repeated 12-element sequence, except block 18, in which elements were pseudo-randomly organized (random succession excluding immediate repetition of the same position). Participants were not informed about the sequential nature of the material. Then, depending on their assigned condition, they were informed either that they were allowed to return home and sleep normally (Regular Sleep (RS) condition), or that they had to spend the night awake in the laboratory (Sleep Deprivation (SD) condition). A night protocol was preferred to a day nap paradigm as naps do not always seem sufficient to improve procedural memory, especially when including NREM sleep only [66]. In the SD condition, participants were allowed quiet activities (e.g., watching non-arousing movies or playing board games) under supervision of the experimenter. Typing on a keyboard was forbidden to prevent motor interferences. Isocaloric food portions and water ad libitum were available all night. Every hour, participants answered the Karolinska Sleepiness Scale [67] to document their sleepiness [68] and performed the 10-min version of the Psychomotor Vigilance Task (PVT [69]) every 2 h. Before returning home, they were instructed to attempt not to sleep before the evening to stay in line with their circadian rhythm; a short nap (max 2 h) after lunch was allowed if really needed by the participant. In both RS and SD conditions in the morning of Day 2, half of the participants had to perform 2 sequential blocks between 8 and 8:30 am after the experimental night (Reactivation condition); the other half did not perform the SRT task that day (No Reactivation condition). Participants spent the 3 following nights at home as one night of SD was shown to impact sleep features up to 2 nights following the SD night [70]. At Day 5, all participants returned to the lab in the late afternoon at the same time as the learning session to perform a final retest (2 sequential blocks; see Figure 1). A 5 min version of the PVT [71] was also administered before each SRTT session to control for changes in behavioural alertness. Most of the participants subjectively reported noticing the sequential nature of the task by the end of the experiment. Hence, 4 groups were constituted crossing two parameters (i.e., Regular Sleep (RS)/Sleep Deprivation (SD) and Morning Reactivation/No Morning Reactivation) in a between-group design. 

### 4.3. Motor Task

**Figure 5:**
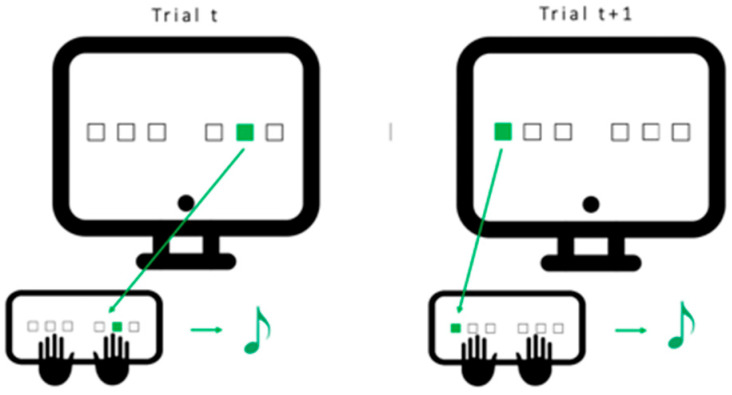
*Serial Reaction Time Task (SRTT):* Participants are seated in front of the computer with 6 fingers (no pinkies and thumbs) placed on the 6 keys matching the 6 positions on the screen. Each time one of the positions lights up, the participant is instructed to press the matching key as fast and as accurately as possible. Once the key is pressed, an auditory tone matching this key is played and the next cue is presented after 500 msec. One block consists of 96 trials (either sequential or pseudo-random).

Our SRTT [48] version is a 6-choice reaction time task coupled with auditory tones (see Figure 5) running on PsychoPy3 v2020.2.10 (Nottingham, UK). Coupling with auditory tones was performed for the sake of a future experiment. Participants were seated in front of a computer screen with 6 fingers (index, middle and ring fingers of each hand) positioned on 6 response keys, each located below one of 6 positions horizontally arranged on the screen. During the task, a visual cue appeared at one of the 6 positions on the screen, and the participant had to press the corresponding response key as fast and accurately as possible. Once the key was pressed, an auditory sound matching this key/position was emitted, and a new cue was presented after a 500 msec delay. One block was composed of 96 trials, followed by a short rest period (rest duration self-defined by the participant). The 96 trials in each block were either constituted of a repeated 12-element sequence (5-3-1-6-2-4-1-5-2-3-6-4) or a pseudo-random succession (block 18). 

### 4.4. Performance Assessment and Analyses

SRTT performance was computed for each block using mean reaction time (RT) and accuracy (number of correct triplets throughout the 96 keypresses, as humans show a natural tendency to divide behavioural sequences in chunks [72,73] up to 3 elements [74]). Both frequentist and Bayesian statistics were performed in JASP 0.15.1 (JASP Team, 2022; https://jasp-stats.org/ (accessed on 15 January 2023)). Bayesian factor [BF] values > 3 are strongly supportive of the H1 hypothesis for a difference between conditions, BF < 0.33 are strongly in favour of the null hypothesis, and 0.33 < BF < 3 are deemed inconclusive [75]. Welch *t*-tests or Welch ANOVAs were preferred to Student *t*-tests or regular ANOVAs when homogeneity of variance was violated. Mann–Whitney U-tests were applied instead of Student *t*-tests when normality was violated. Degrees of freedom were corrected with Greenhouse–Geisser sphericity correction when Mauchly’s sphericity test indicated violated assumption. Bonferroni correction for multiple comparison was applied when post hoc tests were conducted. All tests are based on a two-sided significance level set a *p* < 0.05.

## Data Availability

Available on demand.
